# Contributing Factors to Burnout in Healthcare Professionals—Does Emotional Intelligence Play a Protective Role? A Narrative Review

**DOI:** 10.3390/healthcare13172156

**Published:** 2025-08-29

**Authors:** Ioana Ruxandra Stoian-Bălăşoiu, Liliana Veronica Diaconescu, Alexandra Ioana Mihăilescu, Sabina Stan, Adela Magdalena Ciobanu, Ovidiu Popa-Velea

**Affiliations:** 1 Department of Medical Psychology, Faculty of Medicine, “Carol Davila” University of Medicine and Pharmacy, 020021 Bucharest, Romania; ioana-ruxandra.stoian@drd.umfcd.ro (I.R.S.-B.); ovidiu.popa-velea@umfcd.ro (O.P.-V.); 2 Department of Psychiatry, “Prof. Dr. Alexandru Obregia” Clinical Hospital of Psychiatry, 041914 Bucharest, Romania; adela.ciobanu@umfcd.ro; 3 Department of Special Psychopedagogy, Faculty of Psychology and Educational Sciences, University of Bucharest, 050663 Bucharest, Romania; sabina.stan@fpse.unibuc.ro; 4 Neuroscience Department, Discipline of Psychiatry, Faculty of Medicine, “Carol Davila” University of Medicine and Pharmacy, 020021 Bucharest, Romania

**Keywords:** burnout, emotional intelligence, healthcare professionals, occupational well-being

## Abstract

**Background**: In light of the concerning increase in burnout among healthcare professionals, it is essential to identify the specific factors that contribute to this phenomenon and can be addressed. This narrative review synthesizes evidence on the relationship between burnout and emotional intelligence (EI) among healthcare professionals, alongside additional factors that may influence both concepts. **Methods**: A structured search in OVID, PubMed, Medline, Scopus, and Web of Science (2000–2024) was conducted. The inclusion criteria were English language and peer-reviewed studies assessing both burnout and EI in healthcare professionals. The exclusion criteria were non-English papers, studies without EI–burnout correlation, or involving non-healthcare populations. Thirty-one eligible studies were included in this analysis. **Results**: The findings suggest a consistent inverse correlation between EI and burnout across various healthcare professionals, including doctors, nurses, and residents. Higher EI was associated with reduced levels of emotional exhaustion and depersonalization and a greater sense of personal accomplishment. Burnout was found to be prevalent among younger healthcare workers, particularly residents, with contributing factors including exposure to workplace violence, high workload, and diminished psychological ownership. In contrast, associations that suggest protective influences on emotional intelligence included spiritual intelligence, self-control, income, and healthy habits, such as sufficient sleep and physical activity. **Conclusions**: This narrative review highlights a consistent inverse association between EI and burnout in healthcare professionals. Given that both burnout and EI are affected by adjustable individual and organizational elements, specific interventions aimed at enhancing EI and improving workplace conditions may provide effective techniques to boost clinician occupational well-being and performance.

## 1. Introduction

In recent years, the prevalence of burnout syndrome among healthcare workers has sharply increased, estimated to be double that reported in the general population [1]. According to the recent literature, half of the investigated doctors technically meet the criteria for burnout [2], even if they do not complain or act as professionally impaired.

Maslach and Leiter consider burnout as a „breakdown in the relationship of people and their work” [3], which occurs as a psychological syndrome response to the exposure to chronic personal and interpersonal stressors associated with the job. Although it does not constitute a disease, the profound impact this psychological phenomenon has on the individual makes it essential to take into consideration when evaluating the development of all aspects of one’s life. It includes three dimensions:

1. Emotional exhaustion (EE), perceived by the individual as an emotional depletion, as if „their reserves are consumed” [1];
2. Depersonalization (DP), characterized by a subjective feeling of detachment and even diminished empathy [4];
3. Decreased sense of personal accomplishment (PA), which can distort one’s self-evaluation of their work and leads them to feel less competent [5].

The consequences of burnout can influence both physical and mental health, and they might vary from muscle tension, sleep disorders, anxiety, cognitive dysfunction, social disconnection, or depression [6]. As a whole, burnout and its outcomes can surpass the professional level and significantly decrease the overall functionality and well-being. Sometimes, burnout evolution can go as far as to contribute to the development of a serious condition, making it rather an extensive problem than a syndrome in itself. Moreover, burnout can be self-perpetuated by inadequate coping strategies and reluctance in seeking support from others, possibly accentuating the stigma around seeking help [7,8].

In the long term, all these effects can influence not only health professionals but also their patients and families. Possible mechanisms involved include, for example, the higher risk of taking careless decisions, poor communication, and an overall decline in their professional performance [9].

Since burnout has such an impact on a great array of domains in an individual’s life and because it can have such a great toll on how one’s work is perceived and influenced, it becomes increasingly important to identify the variables contributing to its occurrence. They include individual and organizational factors. Specific individual factors comprise the presence of certain individual traits, such as high neuroticism and low resilience, alexithymia, and perfectionism [10,11], alongside maladaptive coping mechanisms, such as avoidance, denial, substance use, and a lack of emotional regulation [7]. A distinct category of individual factors is represented by demographic variables (e.g., age and gender), with lower age and female gender being correlated with a higher predisposition to burnout, an effect which could be due to lower resilience [12].

When it comes to the organizational factors that predispose individuals to burnout, a meta-analysis conducted by Bria et al. [13] concluded that perceived stress, high workload, and the imbalance between personal and professional life were the most frequently identified contributors. In addition, the level of work experience, core organizational values, and perceived recognition of work were also identified as key environmental variables in the development of burnout [14,15,16].

In regard to protective factors, a meta-analysis conducted by de Mendonça et al. [17] indicated that higher emotional intelligence (EI) can play a protective role against burnout risk, with similar effects observed from mindfulness, stress-management strategies, time-management skills, and self-care practices [18,19]. Organizational factors preventing or delaying burnout include work–life balance, meaningful work, a positive workplace culture, and good-quality relationships with colleagues and superiors [20,21]. Among the abovementioned factors, EI plays a distinctive role, as it has not only a protective role against burnout, but also a significant positive effect on overall well-being and on the wise use of stress-management skills [22]. Accordingly, EI is a concept that has triggered a lot of debate and has been studied consistently in relation to the individual capacities to adapt and succeed in social environments [23].

The concept of EI can be considered either a set of abilities or a set of personality traits, referring to one’s capacity to understand, express, and regulate one’s own or others’ emotions [24]. Literature data point out that individuals who are able to experience emotions with clarity and who can process them effectively are more efficient at applying mood-regulation strategies. These skills transcend individual use and can be applied to interpersonal relationships [25]. When the management of emotions is put into action, the interacting experience becomes a more empathetic one [26]. According to the model structured by Goleman [27], EI is associated with five major personality traits:

1. Self-awareness represents an individual’s ability to recognize their own emotions, as well as the impact of those emotions on other people;
2. Self-management (or self-regulation) assumes the recognition of negative emotions, as well as controlling and forwarding them toward a more productive purpose;
3. Inner motivation represents the tendency to be driven by values and passion, not only by external reward;
4. Social awareness includes the ability to manage and guide the relationships with others;
5. Empathy represents the capacity to understand and share the feelings of others [24,28].

The consistent study of EI in the current literature and the rise in the popularity of the concept itself are challenged by a number of difficulties:

- A methodological heterogeneity in approach, making it more complicated to reach a consensus [24,29];
- Diversity when it comes to cultural and contextual differences regarding work ethic, work–life balance, or the approach of expressing feelings in certain regions. In the context of obtaining most of the data used by self-administered questionnaires, the cultural stigma or bias may be important contributors to the accuracy of data. The individualistic vs. collectivist models can also shape the way not only work is perceived, but also how individuals tend to seek or receive support and guidance [30].

Regarding challenges in understanding how exactly EI influences burnout, the exact mechanism is still unclear. According to Glenn [31], EI could improve coping strategies and emotional regulation, enhance the use of social support, and even influence stress-response, but the precise underlying mechanisms are yet unknown. Furthermore, the multitude of individual psychological factors associated with EI and their interdependence increases the difficulty of pinpointing a specific pattern. According to the Job–Demands–Resources model (JD-R) of burnout, EI can act as a personal resource that regulates the demands of the job that contribute to burnout and buffers their personal impact, alongside building resilience. In other words, although EI does not change or eliminate job demands, it better equips the individual against these adversities [32]. According to Hobfoll et al., the Conservation of Resources Theory, higher EI levels act as a personal resource that could mediate the loss of individual resources that occurs in burnout, by offering the individual better skills to both prevent resource loss and further gain additional resources, employing techniques like emotional regulation, building social support, and developing alternative coping mechanisms [33].

According to a study conducted by Di Gesto et al., psychological inflexibility and difficulties in emotional regulation may play a crucial role when it comes to the levels of perceived stress, meaning that employees who displayed difficulties in regulating emotions were more prone to display psychological inflexibility, which, furthermore, had an impact on stress perception [34].

When focusing on healthcare workers, better EI skills should result in better communication, increased empathy toward the patients and their families, and, therefore, a better therapeutic relationship [35]. However, when it comes to applying the same skills to oneself, it is debatable if having better-developed emotional skills is efficient in delaying or even preventing the effects of chronic occupational stress. Furthermore, one’s ability to regulate their emotions could also be impacted by various factors, such as the leadership style they work under, workload, and exposure to suffering. The gap in the existing literature is related to the limited understanding of how different factors, especially emotional intelligence, interact to influence the development of burnout among healthcare workers and the lack of evidence-based strategies to improve protection against these risk factors.

This study aims to investigate the relationship between EI and burnout in healthcare workers following three research questions (RQ):

RQ 1: What is the burnout prevalence among healthcare workers, and what are the main contributing factors to burnout?

RQ 2: Does EI play a role in protecting against burnout?

RQ 3: Which additional factors influence EI in healthcare workers?

## 2. Materials and Methods

The present work is a narrative review, with its reporting quality checked against the SANRA (Scale for the Assessment of Narrative Review Articles) criteria, with all items addressed to ensure methodological transparency and rigor. A narrative approach was chosen due to the heterogeneity of study designs, populations, and instruments used to assess EI and burnout, which made a systematic synthesis inappropriate but facilitated a comprehensive mapping and interpretative integration of the evidence on the relationship between healthcare workers’ EI and burnout while also identifying additional modulating factors relevant for targeted interventions.

A structured literature search that focused on the topics of EI and burnout in healthcare workers was conducted in OVID, PubMed, Medline, Scopus, and Web of Science databases for English-language studies published between January 2000 and April 2024. Search terms combined “emotional intelligence” AND (“burnout” OR “the burnout syndrome”) IN (“healthcare workers” OR “healthcare professionals” OR “doctors” OR “nurses” OR “physician” OR “residents”), using Boolean operators and filters for language and time frame. The last search was conducted on 17 February 2025.

Inclusion criteria were peer-reviewed studies in English, focused on healthcare professionals, and that investigated the correlation between EI and burnout.

The exclusion criteria were represented by studies focused on students or personnel who did not work in a hospital and veterinarians, centered only on a limited number of burnout components (such as emotional exhaustion or depersonalization), or those that did not correlate EI to burnout. To enhance accuracy, only peer-reviewed articles were included; the grey literature (e.g., conference proceedings, preprints, dissertations) was excluded.

**Figure 1:**
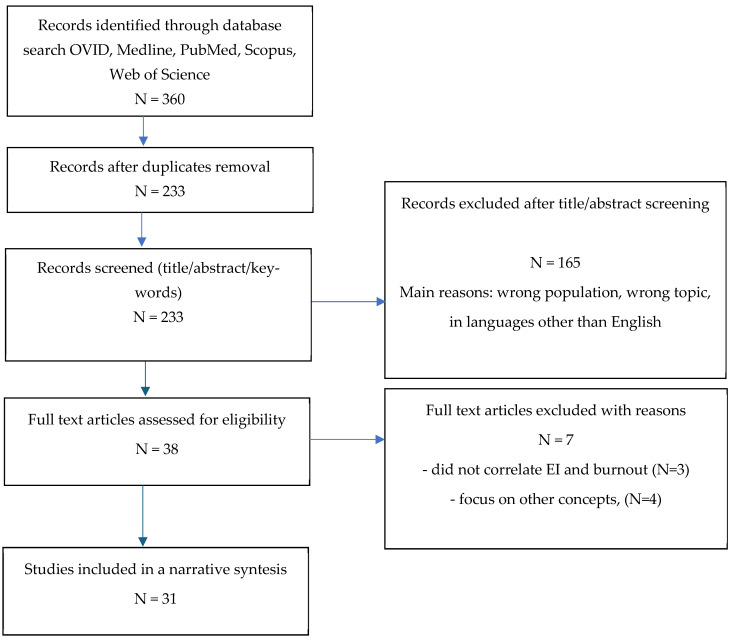
Flow diagram illustrating study selection process.

Two researchers independently screened titles, abstracts, and full texts and extracted study data, with disagreements resolved through discussion. This process is summarized in Figure 1 (PRISMA-like format for transparency).

Titles and abstracts were evaluated to eliminate irrelevant papers (e.g., non-English publications, research involving non-healthcare populations—veterinarians, teachers, administrative staff, medical students, or those not addressing both emotional intelligence and burnout). Full texts were examined to verify eligibility. To improve conceptual clarity, we excluded studies examining only single components of burnout (e.g., emotional exhaustion) or emotional intelligence (EI). Since some of the articles also studied additional factors both in correlation to EI and burnout, the narrative approach allowed us to also incorporate this additional information.

The first search yielded 360 hits; after deduplication, 233 titles/abstracts were screened, and 165 were excluded. Of 38 full texts reviewed, 7 were excluded for not meeting inclusion criteria (reasons detailed in Supplementary Table S1), resulting in 31 studies included in the final synthesis.

## 3. Results

### 3.1. General Findings

Table 1 shows a summary of the study’s characteristics, instruments, and principal findings.

All 31 articles presented in Table 1 measured EI, burnout, and the correlation between them. Sample sizes varied from 28 to 12,704 respondents, covering a large cultural background and demographical diversity. Eleven articles were specifically focused on resident doctors; eight articles focused on nurses, and five on doctors. Seven articles studied healthcare workers with different professions combined (doctors, nurses, interns, physical therapists, and other caregivers). The comparatively high number of studies centered on residents illustrates their depiction in the current literature as one of the most predisposed categories to burnout.

All identified articles were published within the last 15 years, which could also indicate an increasing interest in the study of EI potential benefits, on the one hand, and the increasing burnout prevalence in healthcare workers, on the other hand.

Across all identified articles, burnout syndrome was consistently understood as an occupational phenomenon, resulting from the prolonged exposure to work-related stress, while EI was seen as the ability to recognize, understand, manage, and even change individual emotions or the emotions of others.

Given the great variability in both EI and burnout instruments, we acknowledge that this might impact the association between the two concepts by slightly influencing the conceptual framework of the already subjective variables.

#### Measurement and Interpretation of Constructs

**Burnout.** When it comes to measuring burnout levels, most of the identified studies (22) opted for using The Maslach Burnout Inventory (MBI); studies used either the **MBI-HSS** (human services; domains: Emotional Exhaustion [EE], Depersonalization [DP], Personal Accomplishment [PA]) or the **MBI-GS** (general survey; domains: Exhaustion, Cynicism, and Professional Efficacy). Importantly, higher PA/Professional Efficacy reflects *lower* burnout; higher EE/DP (or Exhaustion/Cynicism) reflects *higher* burnout. Although both scales employ a three-dimensional approach to burnout, the MBI-HSS specifically targets helping professions preferring depersonalization as a domain, rather than cynicism, which would be more tailored for this professional category than the general population. Because included studies used non-equivalent instruments, we interpreted scores within the instrument rather than pooling raw ranges.

We also included studies using the Brief Burnout Questionnaire (CBB), which also approaches burnout as a three-dimensional concept, in a short and accessible form; The Tedium Index, which is an instrument rather focusing on exhaustion as a unidimensional element; The Questionnaire for the Assessment of Occupational Burnout Syndrome (CESQT), which comprises four subscales—enthusiasm toward work, psychological exhaustion, indolence and brings as a novelty element the concept of guilt, comparing to other tools; The Link Burnout Questionnaire (LBQ), which also structures burnout as a four-dimensional concept, comprising psychological exhaustion, relationship deterioration, sense of failure and psychosomatic complaints; The Oldenburg Burnout inventory (OBLI), containing two core dimensions, exhaustion and disengagement; and the Shirom-Melamed Burnout Measure (SMBM), which views burnout as a three-dimensional concept focused around exhaustion: physical fatigue, emotional exhaustion and cognitive weariness. This diversity reflects, in part, the various conceptualizations of the burnout syndrome, which resulted in a multitude of measurement instruments that overlap to some extent [36]. Accordingly, results are summarized per instrument and domain, avoiding cross-tool pooling.

**Emotional intelligence (EI).** We classify EI measures as (a) **ability-based** tests (e.g., MSCEIT) that score performance on emotion tasks, and (b) **trait/self-report** measures (e.g., TEIQue/TEIQue-SF, WLEIS, SSEIT, EIS). Although the Mayer–Salovey–Caruso Emotional Intelligence Test (MSCEIT) is a standard ability-based measure, none of the included studies employed it. All studies in this review used The Trait Emotional Intelligence Questionnaire (TEIQ) (13 studies) or its short form, TEIQ-SF, which measures self-reported emotional traits, like emotion regulation and self-awareness, followed by the use of The Emotional Intelligence Scale (EIS), which evaluates skills like emotion perception or social skills. The other instruments used to determine the levels of EI were The Schutte Self-Report Emotional Intelligence Test (SSEIT), which is based in the Mayer–Salovey model, in a self-report format; The Brief Inventory of Emotional Intelligence for Adults (EQ-i-20M), which is more frequently used in clinical settings and in the workplace; The Trait Meta-Mood Scale 24 (TTMS-24), which distincts itself by focusing on the metacognitive component of emotions; The Swinburne University Emotional Intelligence Test, which evaluates empathy, emotion management, and social skills; The Nurses Job Emotions Scale (NJES), used to evaluate job-specific emotions; The Emotional Quotient Test, based on the mixed-models of EI; The Emotional Intelligence Questionnaire of Cyber or sharing EI, which takes into consideration EI in digital interactions; The Scale of Emotional Functioning: Health Service Provider, focusing on EI in healthcare providers; and The Wong and Law Emotional Intelligence Scale (WLEIS), which comprises four dimensions: self-emotional appraisal, others’ emotion appraisal, use of emotion, and emotional regulation. Trait EI instruments often report facets, such as **self-emotions appraisal, others’ emotion appraisal, use of emotions, and regulation**, or broader domains such as well-being, self-control, emotionality, and sociability. Where reported, we extracted facet-level results to reduce over-generalization. According to a review conducted by Bru-Luna et al. [37], there has been an increase in the number of instruments used to measure EI, with over 40 scales available at the time being. This reflects both the increasing interest in the concept and its utility, but also the difficulty of constructing objective criteria to measure EI.

**Table 1:** Characteristics of the 31 studies included in the narrative review of country of origin, study design, participant characteristics, EI, and burnout measurement tools; main findings are for all included studies.

No.	Study Title	Authors, Position in Reference List	Year	Country	Study Design	Sample Size	Type of Personnel	Instruments Used for Measuring Burnout	Instruments Used for Measuring EI	Main Findings		
										Correlations Between Burnout and EI	Factors Contributing to Burnout	Modulating Factors for EI
1	The Influence of Emotional Intelligence on Job Burnout of Healthcare Workers and Mediating Role of Workplace Violence: A Cross-Sectional Study	Cao, Y., Gao, L., Fan, L., Jiao, M., Li, Y., & Ma, Y. [38]	2022	China	Cross-sectional	2061	Physicians, nurses, and medical technicians	MBI-GS	EIS	EI was significantly negatively associated with all three dimensions of job burnout	Exposure to violence is correlated with higher burnout	Female gender significantly correlated with higher EI
2	Effect of spiritual intelligence, emotional intelligence, psychological ownership, and burnout on caring behaviour of nurses: a cross-sectional study	Kaur, D., Sambasivan, M., Kumar, N. [39]	2013	Malaysia	Cross-sectional	550	Nurses	MBI-HSS	SSEIT	Nurses with higher levels of EI suffer lower levels of burnout	Psychological ownership correlates with lower levels of burnout	Spiritual intelligence influences EI
3	Association of Burnout With Emotional Intelligence and Personality in Surgical Residents: Can We Predict Who Is Most at Risk?	Lindeman, B., Petrusa, E., McKinley, S., Hashimoto, D. A., Gee, D., Smink, D. S., … & Phitayakorn, R. [40]	2017	United States	Longitudinal cohort	143	Resident doctors	MBI	TEIQ-SF	Burnout scores were significantly inversely correlated with all 4 facets of EI and EI total	Female sex and older age are associated with higher burnout levels. Agreeableness and positive work experiences were inversely correlated with burnout	EI global scores decrease significantly with age
4	Burnout is Associated With Emotional Intelligence but not Traditional Job Performance Measurements in Surgical Residents	Cofer, K. D., Hollis, R. H., Goss, L., Morris, M. S., Porterfield, J. R., Chu, D. I. [41]	2018	United States	Cross-sectional	40	Resident doctors	MBI-HSS	TEIQ-SF	The mean EI scores were lower in residents with burnout than in residents without burnout		
5	The Contribution of Emotional Intelligence to the Components of Burnout: The Case of Health Care Sector Professionals	Ünal, Z [42]	2014	Turkey	Cross-sectional	136	Trainees, interns, nurses, and doctors	MBI	EIS	EI has a positive contribution to PA but negative contributions to EE and DP	Married individuals report lower levels of burnout	
6	Burnout Risk and Protection Factors in Certified Nursing Aides	Molero Jurado, M. D. M., Pérez-Fuentes, M. D.C., Gázquez Linares, J.J.G., Simón Márquez, M. D.M., Martos Martínez, Á. [43]	2018	Spain	Cross-sectional	278	Nurses	CBB	EQ-i-20M	Burnout Syndrome score is significantly related negatively to all the EI factors	Lower age is linked to a higher burnout risk; the group of professionals with a permanent contract showed a significantly higher mean score in burnout	
7	Exploring the relationship between burnout and emotional intelligence among academics and clinicians at King Saud University	Almeneessier, A.S., Azer, S. A. [44]	2023	Saudi Arabia	Cross-sectional	126	Medical academics, doctors	MBI-HSS	TEIQ-SF	An inverse interrelation was reported between burnout and EI	Workplace risk factors for burnout: workload, job control, lack of a supportive environment, recognition and rewards, equitability, and organizational values	In contrast with other studies, we reported that EI and its elements were higher in men than in women, which can be explained by cultural differences
8	Emotional intelligence, workplace conflict, and job burnout among critical care physicians: a mediation analysis with a cross-sectional study design in Egypt	Kasemy, Z. A., Sharif, A. F., Bahgat, N.M., Abdelsattar, S., Latif, A.A. A. [45]	2023	Egypt	Cross-sectional	144	Doctors	MBI	TEIQ	EI could significantly predict job burnout across various dimensions, and it showed a negative association with EE, depersonalisation, and reduced personal achievement.	Age and years of experience, conflict management skills, and CoQ10 levels have a negative correlation with burnout. Exposure to violence and inadequate resources and facilities had a positive correlation with burnout levels	Conflict management and CoQ10 levels exhibited positive correlations with EI
9	The Effect of Emotional Intelligence on Burnout in Healthcare Professionals	Năstasă, L. E., Fărcaş, A. D. [46]	2015	Romania	Cross-sectional	120	Doctors and nurses	MBI	EIS	The level of burnout experienced by the medical personnel did not correlate with the level of EI development.	The burnout syndrome is felt more acutely by women	
10	Analysis of the Risk and Protective Roles of Work-Related and Individual Variables in Burnout Syndrome in Nurses	Pérez-Fuentes, M. D.C., Molero Jurado, M. D.M., Martos Martínez, Á., Gázquez Linares, J. J. [47]	2019	Spain	Cross-sectional	1236	Nurses	CBB	EQ-I-M20	There was a negative association between burnout and various factors of EI	Spending more time with colleagues and patients and reporting good-quality relationships exhibits a negative relationship with burnout. Nurses with permanent contracts had a higher mean score for burnout than those on temporary contracts	
11	Emotional Intelligence in Internal Medicine Residents: Educational Implications for Clinical Performance and Burnout	Satterfield, J., Swenson, S., Rabow, M. [48]	2010	United States	Cross-sectional	28	Resident doctors	The Tedium Index	EIS	EI scores increased over the course of an academic year, and higher year-end scores correlated with less burnout and higher overall clinical performance and interviewing ratings.		
12	Effect of Emotional Intelligence and Psychosocial Risks on Burnout, Job Satisfaction, and Nurses’ Health during the COVID-19 Pandemic	Soto-Rubio, A., Giménez-Espert, M.D.C., Prado-Gascó, V. [49]	2020	Spain	Cross-sectional	125	Nurses	CESQT	TMMS-24	The emotional repair component stands out as an element of EI that should be enhanced to prevent the possible adverse effects of psychosocial risks on nurses, specifically those related to burnout, psychosomatic complaints, and job satisfaction.		
13	Emotional Intelligence and Burnout in Surgical Residents: A 5-Year Study	Gleason, F., Baker, S.J., Wood, T., Wood, L., Hollis, R.H., Chu, D.I., Lindeman, B. [50]	2020	United States	Longitudinal cohort	236	Resident doctors	MBI	TEIQ-SF	Burnout scores showed significant inverse correlation with the 4 domains of EI, total job resources score, and all 4 sub-domains of job resources.	Individuals who were subjected to disruptive behaviors (particularly others taking credit for work and public humiliation) were more likely to experience higher burnout levels. Each additional PGY year demonstrated an incremental increase in burnout	
14	Relationship of Emotional Intelligence and Burnout among MBBS Doctors of Himachal Pradesh	Kaul, I., Reddy, K.J. [51]	2022	India	Cross-sectional	190	Doctors	MBI	SSEIT	A highly negative correlation is found between EI, EE, and DP, and a positive correlation between EI and PA		
15	The Relationship between Burnout Syndrome and Emotional Intelligence in Healthcare Professionals	Vlachou, E. M., Damigos, D., Lyrakos, G., Chanopoulos, K., Kosmidis, G., Karavis, M. [52]	2016	Greece	Cross-sectional	148	Doctors, nurses, and physical therapists	MBI	TEIQ-SF	There is a positive relationship between EI and Burnout syndrome, as EI acts protectively against Burnout syndrome and even reduces it		
16	The role of Emotional Intelligence in healthcare professionals’ burnout	Arnone, R., Cascio, M.I., Parenti, I. [53]	2019	Italy	Cross-sectional	148	Doctors, nurses, and other caregivers	LBQ	SSEIT	There is a negative and significant correlation between Burnout and EI		
17	Emotional intelligence as a moderator in the stress–burnout relationship: a questionnaire study on nurses	Görgens-Ekermans, G., Brand, T. [54]	2012	South Africa	Cross-sectional	122	Nurses	MBI	The Swinburne University Emotional Intelligence Test	Consistent inverse relationships between emotional control and management as dimensions of EI, and stress and burnout emerged. A differential effect of high vs. low EI on the stress–burnout relationship was evident.	Workload and the work/family interface emerged as significant predictors of burnout	
18	Emotional Intelligence Buffers the Effects of Negative Emotions on Job Burnout in Nursing	Szczygiel, D. D., Mikolajczak, M. [55]	2018	Poland	Cross-sectional	188	Nurses	OLBI	NJES	Negative emotions do not always lead to burnout, but they particularly do for nurses who lack EI.		
19	The Role of Emotional Intelligence on Health Care Professionals’ Occupational Stress and Burnout	Tiwari, S., Bhagat, D. [56]	2020	India	Cross-sectional	388	Nurses and Doctors	OLBI	Emotional Quotient Test	The dimensions of EI, emotional sensitivity, emotional maturity, and emotional competency have been reported to significantly predict all seven dimensions of occupational stress and the dimensions of burnout, and the order and strength of the predictors differ across the two groups of healthcare professionals.	A positive relationship was found between age, working experience, and stress, with younger healthcare professionals and those with a shorter length of service experiencing more stress	EI levels increased with the age and experience of the respondents on emotional intelligence
20	The Effect of Emotional Intelligence and Job Stress on Burnout: A Structural Equation Model among Hospital Nurses	Samaei, S.E., Khosravi, Y., Heravizadeh, O., Ahangar, H. G., Pourshariati, F., Amrollahi, M. [57]	2017	Iran	Cross-sectional	300	Nurses	MBI-HSS	The Emotional Intelligence Questionnaire of Cyber or sharing-EI	There was meaningful relationship between EI and job stress with nurses’ occupational burnout. The EI was effective on job stress		A significant difference was found in correlation with income–the higher income correlated with increasing EI levels
21	Evaluating and Exploring Variations in Surgical Resident Emotional Intelligence and Burnout	Beierle, S.P., Kirkpatrick, B.A., Heidel, R.E., Russ, A., Ramshaw, B., McCallum, R.S., Lewis, J.M. [58]	2019	United States	Longitudinal cohort	86	Resident doctors	MBI	Scale of Emotional Functioning: Health Service Provider	The data confirm an inverse relationship between EI and burnout		
22	The Mediating Role of Emotion Management, Self-Efficacy, and Emotional Intelligence in Clinical Nurses Related to Negative Psychology and Burnout	Yu, C., Liu, Z., Zhao, M., Liu, Y., Zhang, Y., Lin, A., … & Wan, H. [59]	2023	China	Cross-sectional	12,704	Nurses	MBI-GS	ETS	EI among nurses could reduce the incidence of burnout		
23	Emotional intelligence, perceived stress, and burnout among resident doctors: An assessment of the relationship	Swami, M. K., Mathur, D.M., Pushp, B.K. [60]	2013	India	Cross-sectional	56	Resident doctors	SMBM	TEIQ	There is a significant negative correlation between burnout and trait EI and a positive correlation with perceived stress, indicating that burnout is probably influenced by perception of stress and EI.		
24	Associations between emotional intelligence and doctor burnout, job satisfaction, and patient satisfaction	Weng, H.C., Hung, C.M., Liu, Y.T., Cheng, Y.J., Yen, C.Y., Chang, C.C., Huang, C.K. [61]	2011	Taiwan	Observa-tional	110	Doctors	MBI	WLEIS	Higher EI was significantly associated with less burnout and higher job satisfaction. In addition, less burnout was not only associated with higher levels of patient satisfaction but also with higher levels of job satisfaction.		
25	Correlation among Perceived Stress, Emotional Intelligence, and Burnout of Resident Doctors in a Medical College of West Bengal	Mitra, S., Sarkar, A.P., Haldar, D., Saren, A.B., Lo, S., Sarkar, G.N. [62]	2018	India	Cross-sectional	63	Resident doctors	SMBM	TEIQ	Burnout had a significant positive correlation with perceived stress and negative correlation with EI-well-being and positive correlation with EI-self-control and sociability.		
26	Self-control as mediator between emotional intelligence and burnout among doctors	Jahanzeb, Z., Parveen, S., & Khizar, U. [63]	2023	Pakistan	Quantita-tive	150	Doctors	MBI	EIS	EI is negatively correlated with burnout. The result showed a significant negative correlation between burnout and self-control.	Burnout appears to be a more female experience, with women reporting it at a higher rate than men	
27	Emotional Intelligence and Burnout in Plastic Surgery Residents: Is There a Relationship?	Bin Dahmash, A.B., Alhadlaq, A.S., Alhujayri, A.K., Alkholaiwi, F., Alosaimi, N.A. [64]	2019	Saudi Arabia	Cross-sectional	37	Resident doctors	MBI	TEIQ-SF	There is a positive correlation between higher levels of EI and sense of personal achievement, whereas a negative correlation was observed between higher levels of EI and EE and DP among the residents in this study.	Significant risk factors for burnout included dissatisfaction with plastic surgery as a career choice, dissatisfaction with income, and dissatisfaction with the role in the operating room	
28	Emotional Intelligence, Burnout, and Wellbeing Among Residents as a Result of the COVID-19 Pandemic	Kirkpatrick, H., Wasfie, T., Laykova, A., Barber, K., Hella, J., Vogel, M. [65]	2022	United States	Cross-sectional	81	Resident doctors	MBI	TEIQ-SF	EI continues to partially protect our residents’ burnout and well-being.		The COVID-19 pandemic’s initial surge appeared to negatively alter the protective effect of residents’ emotional intelligence on their burnout and well-being in our community hospital.
29	Burnout and well-being of medical and surgical residents in relation to emotional intelligence: A 3-year study	Wasfie, T., Kirkpatrick, H., Barber, K., Hella, J., Lange, M., Vogel, M. [66]	2024	United States	Longitudinal	77	Resident doctors	MBI	TEIQ-SF	EI was inversely related to burnout and distress and is related to wellness factors in this community–hospital–based resident study sample.		
30	Emotional Intelligence and Burnout Among Otorhinolaryngology–Head and Neck Surgery Residents	Sharaf, A. M., Abdulla, I.H., Alnatheer, A.M., Alahmari, A.N., Alwhibi, O.A., Alabduljabbar, Z., … & Alkholaiwi, F.M. [67]	2022	Saudi Arabia	Cross-sectional	51	Resident doctors	MBI	TEIQ-SF	This study showed that surgical specialty residents with higher EI levels had a lower risk of burnout		One extra sleeping hour is associated with a 0.44-unit increase in the average EI score; the average EI score was 0.52 units higher in residents who exercised than in those who did not
31	Longitudinal study of emotional intelligence, well-being, and burnout of surgical and medical residents	Wasfie, T., Kirkpatrick, H., Barber, K., Hella, J. R., Anderson, T., Vogel, M. [68]	2023	United States	Longitudinal	80	Resident doctors	MBI	TEIQ-SF	EI is associated with well-being and burnout in individual residents		

### 3.2. RQ 1: What Is the Burnout Prevalence Among Healthcare Professionals and Which Are the Main Contributing Factors to Burnout?

Despite using a wide variety of instruments and identifying a large range of burnout prevalence, most of the studies—with only two exceptions [39,42]—concluded that burnout represents a substantial problem for healthcare workers. In each of the identified studies, medical personnel were at risk of developing burnout and even presented symptoms in some cases (Table 1). For instance, in the study conducted by Lindeman et al. [40], 51% of the evaluated resident doctors met criteria for high burnout, and only 9% had low levels or no burnout. In the study with the largest sample size [59], 12,704 clinical nurses from 32 general hospitals across China were investigated using the MBI-GS scale, resulting in moderate scores of EE (15.87, SD 6.02), moderate scores of DP (10.80, SD 5.09), and significant scores of decreased PA (25.53, SD 8.65). Overall, they were assessed as displaying moderate to high burnout, with a tendency to experience psychiatric comorbidities in the form of anxiety and depression.

Among MBI-based studies, higher EI consistently aligned with **lower EE/DP (or Exhaustion/Cynicism)** and **higher PA/Professional Efficacy** (i.e., lower burnout). OLBI-based studies converged on **lower Exhaustion/Disengagement** with higher EI; SMBM-based studies showed **lower Physical Fatigue/Emotional Exhaustion/Cognitive Weariness** at higher EI. Because thresholds and scaling differ across tools, we report direction and domain rather than pooled raw ranges.

Since 16 of the identified articles used the Maslach Burnout Inventory, we analyzed their comparative results. Across them, EE ranged from 3.41 to 31.24 (a score of less than 18 means low EE, and over 27 signifies high levels of EE). DP levels varied from 2.2 to 11.9 (scores lower than 5 meaning low levels of DP, and above 10 indicating higher levels of DP). PA ranged from 2.84 to 38.13 (levels under 33 mean high levels of PA, and levels over 40 mean low PA). In MBI-type instruments, higher PA/Professional Efficacy indicates greater accomplishment and *lower* burnout, whereas higher EE/DP (or Exhaustion/Cynicism) indicates *higher* burnout.

Several studies identified additional factors involved in burnout development. By categories, they were depicted as follows:

(a) demographic factors (age and gender);
(b) psychological variables (e.g., psychological ownership and agreeableness);
(c) physiological factors (e.g., coenzyme Q10–CoQ10 levels);
(d) environmental variables (exposure to violence, positive work experience, workload, type of work contract, dissatisfaction with career choices).
(a) Demographic factors

When it comes to gender influence in burnout, several studies [40,46,63] pointed out that higher burnout levels were correlated with female gender.

Regarding age and work experience, Lindeman et al. [40] reported a higher risk of burnout with increased age, whereas other studies [43,45,56] found a negative correlation between age and experience and burnout levels. Sharaf et al. [67] also pointed out that the risk for burnout increased sharply at the beginning of residency training.

(b) Psychological variables

According to the study included in our review by Kaur et al. [39], psychological ownership (PO) among medical personnel creates a sense of responsibility, which influences behavior and was found to have a negative relationship with burnout. The concept of PO represents a state in which an individual feels as though the target of ownership—whether it is a job or an organization—is theirs; therefore, one’s possessions are felt as extensions of the self. PO can mitigate the effects of burnout by giving employees greater autonomy over their work, fostering more meaning in their efforts, and making the entire distressing experience feel more meaningful [69].

Furthermore, in another study included in our review, Lindeman et al. [40] pointed out in their longitudinal cohort that the personal trait of agreeableness was inversely correlated with burnout. The trait of agreeableness refers to a person’s tendency to be considerate towards others, polite, and compassionate.

(c) Physiological factors

A study conducted by Kasemy et al. [45] found a correlation between the functioning of the immune system, as expressed in individual CoQ10 levels, and burnout: higher burnout levels seem to deplete the body’s CoQ10 reserves, therefore making the protective mechanisms weaker. CoQ10 is a vitamin-like compound with roles in energy production and antioxidant protection.

(d) Environmental variables

Ünal [42] identified marital status as a factor contributing to burnout protection, with married individuals reporting lower burnout levels.

Cao et al. [38] identified exposure to violence as a strong burnout predictor, with up to 98% of respondents experiencing verbal violence in the last year, and the personnel exposed to violence displaying higher levels of burnout.

In the longitudinal study conducted by Lindeman et al. [40], positive work experience showed a strong inverse correlation with individual burnout. The magnitude of the relationship for positive work experience was larger than that for negative work experience, proving that an overall positive work experience might have a protective role against burnout in surgical residents. Resident doctors examined in a longitudinal study by Gleason et al. [50] were more prone to burnout if their work environment was aversive and if they were subject to disruptive behaviors, especially when others took credit for their work or exposed them to public humiliation. Dissatisfaction with their career choice, the low income, and the marginal role in the operating room were also factors contributing to resident doctors’ burnout, according to Kirkpatrick et al. [65].

Some studies [43,47] have identified the type of work contract as a contributing factor for burnout development. These authors claim that nurses with a permanent work contract also displayed a higher risk for burnout.

Finally, as expected, high workload was a significant factor contributing to burnout [44,54]. This data is consistent with the findings of other papers not included in this review.

### 3.3. RQ 2: Does Emotional Intelligence Play a Role in Protecting Against Burnout?

With the exception of the study conducted by Năstasă & Fărcaş [46], all the other papers included in this review identified EI as an important contributor in the protection against burnout (Table 1). The concerns mentioned above, despite not identifying a significant correlation between EI and burnout levels in the examined population, still point out a relationship between EI and personal accomplishment in healthcare workers, which, in turn, leads to the authors’ conclusion about the need to develop EI in this work category. As a confirmatory finding, higher EI in the categories of healthcare professionals was negatively associated with burnout by the studies included in this review (Table 1), except for the aforementioned one. Among the studies that employed the most commonly used assessment instrument for burnout, MBI, and the three-dimensional model (EE, DP, and PA), all of them, except the one mentioned before, found that high levels of EI were inversely correlated with EE and DP and directly correlated with PA. In the longitudinal studies identified [58,66,68], higher baseline EI was associated with lower burnout scores at follow-up; however, potential confounding by demographic or occupational factors cannot be ruled out.

### 3.4. RQ 3: Which Additional Factors Influence EI in Healthcare Workers?

Some of the researched studies [38,39,40,67] identified several additional factors significantly related to EI (age, experience, gender, spiritual intelligence, PO, perceived stress levels, income, exercise levels, and sleep hours).

From a longitudinal perspective, PO, spiritual intelligence, and low perceived stress were not only correlated with EI, but also had a significant association with burnout.

When it comes to age and experience influence on EI levels, Lindeman et al. [40] found that EI global scores decreased significantly with increasing age, while Tiwari & Bhagat [56] found a significant positive correlation between EI and age.

In terms of gender, many studies were inconclusive about which gender is more EI-privileged, although the association of gender with EI seemed significant. Cao et al. [38] reported higher EI in women, while Almeneessier et al. [44] found higher EI in men.

Ünal and Molero Jurado et al. [42,43] did not find any correlation of EI to gender or age and explained this finding through other cultural differences rather than feminine stereotypes, potentially obscuring the role of gender.

The study conducted by Tiwari & Bhagat [56] found a positive correlation between EI and income, which could be explained by the EI skills potentially playing a key role in successful careers, especially if they have a higher social exposure.

Sharaf et al. [67] identified an association between EI and healthy daily habits (sleep and exercise). Specifically, they found that the average number of sleeping hours was correlated with the total EI score, and that one extra hour of sleeping a night is associated with a 0.44-unit increase in the average EI score (using the TEIQue-SF). The same study showed that exercise determined a 0.52-unit increase in EI in residents who exercised, compared to those who did not.

## 4. Discussion

The primary goals of this review were (a) to assess the prevalence of burnout in healthcare professionals and highlight the contributing factors to burnout; (b) to identify whether EI provides protection against burnout, and (c) to expose additional variables contributing to EI and which could potentially be subject to change.

### 4.1. Burnout Prevalence and Variability

In terms of burnout prevalence in healthcare professionals, this has been a consistently reported issue, which has additionally grown in interest since the COVID-19 pandemic [70]. In the studies we identified, burnout rates were not always explicitly stated, since most of them focused on the correlations between burnout and predictive variables. However, those who reported burnout prevalence displayed great variability, from low burnout (2.15 on the MBI scale) [39] to high or even alarming burnout, with up to 70% from the maximum values [38]. This great variability can be explained by the heterogeneity of the used instruments and protocols, the substantial array of geographical and cultural backgrounds covered by the included studies, where only a few of them put their conclusions in the context of socioeconomic or income-related frameworks, making the comparison of results across regions difficult, and the use of self-reported questionnaires, which may have induced bias by itself, via the underreporting or overreporting of emotional distress and burnout symptoms.

Amongst the articles that used the MBI, high global burnout scores were mostly associated with high EE, DP, or both. According to Maslach & Leiter [71], EE is considered to be the closest parameter to a conventional stress variable, making it more reliable in predicting burnout-related symptoms.

All the identified articles have been published within the last 15 years, illustrating an obvious increase in interest in the topic. A number of factors could explain this, such as the official recognition of burnout as an “occupational phenomenon” by the World Health Organization [72]. The COVID-19 pandemic may have also played an important role, not only in the increased demands that the medical system had to face, but also in the way that work–life balance has been perceived since [70].

Furthermore, the exposure of mental health issues has recently grown, while the stigma around talking about one’s struggles has steadily decreased. As more and more people come forward to testify about their professional and personal struggles, the need to identify their root causes and address them as early as possible has become imperative [73]. It is also important to keep in mind when analyzing this data that there is great variability in care settings, various specialties, and even the different distribution of resources among various places.

Regarding the gender influence on burnout, the identified studies stated that women were more prone to burnout development. According to a review conducted by Purvanova & Muros [74], these differences stem most probably from higher reporting of burnout by women, specifically when it comes to the EE component. As stated before in the literature, the EE component tends to be considered the most representative for burnout, while, at the same time, it is typically easily identified by women [75]. Self-reporting bias associated with cultural stereotypes surrounding masculine traits may also be involved, with men being less likely to report exhaustion, in order not to be perceived as either weak or unprofessional [76]. To avoid this bias and make future studies more reliable, it could be helpful to use qualitative methods and cross-cultural validation of psychometric instruments assessing burnout and, specifically, EE. Using terminology that is not gender-specific or culture-specific could also assist in reducing the existing research biases.

When it comes to age and work experience, several identified studies [43,45,56,66] found that the lack of experience associated with young age and the beginning of residency was correlated with higher burnout, while, oppositely, Lindeman et al. [40] pointed out that there is a tendency for burnout to increase with age. The first result was supported by a meta-analysis by Brewer & Shapard [77], which concluded that higher burnout levels have been correlated with lower age and less job experience, and by a separate study conducted by Gómez-Urquiza et al. [78], who found that younger age was a significant risk factor for the EE and DP component of burnout and less influential regarding PA. The sharp increase in prevalence of burnout at the beginning of residency may be explained by the multitude of changes in a young professional’s life, such as gaining responsibility, changes in schedule and workload, all the while not yet having the experience to effectively cope with these new challenges. Therefore, it is reasonable to presume that interventions applied at this point in the medical career, targeting resident support and mentorship, are important for addressing this problem and preventing its aggravation.

Factors found as having a protective role against burnout were two-fold: individual (PO, agreeableness, and CoQ10 levels), but also environmental (marital status, exposure to violence, workload, positive work experiences, and dissatisfaction with career choices). Some of these factors (such as workload, PO, and exposure to violence) are circumstantial, so they can be targeted by personalized interventions. A good example is PO, with strategies increasing its leading to higher autonomy, especially by being given decision-making power, promoting hands-on contributions, the use of group symbols, and non-monetary recognition, such as public acknowledgement [79]. These outcomes may not only contribute to a more cohesive work environment and increased personal well-being, but also improve PA scores [80].

Agreeable healthcare professionals tend to reframe patient interaction as more meaningful than draining; they tend to develop better workplace relationships and even have lower rates of conflict due to their prosocial behavior [81]. According to Roberts et al. [82], the idea of personality plasticity implies that certain traits, such as agreeableness, could improve with consistent practice by employing multiple strategies, such as social skill training, reinforcement of the prosocial behavior, perspective taking, and even cognitive intervention techniques. Increasing agreeableness could not only have an impact on individual burnout, but could also have a substantial effect on the therapeutic relationship and perceived quality of care [83].

The decrease in CoQ10 levels as a consequence of exhaustion could be effectively targeted by using nutritional supplements, according to Tsai et al. [84]. The use of CoQ10-only formulations proved to be more effective than CoQ10 compounds, and the longer duration of the treatment had a positive correlation to fatigue reduction. CoQ10 supplementation could, thereby, represent an additional tool, alongside psychological interventions, in the comprehensive targeting of burnout.

Marital status was identified as playing a protective role against burnout development. These findings are consistent with a meta-analysis conducted by Cañadas-De la Fuente et al. [85], who reported that individuals with single or divorced marital statuses are exposed to higher burnout. In contrast, married individuals would benefit from protection against burnout through an array of mechanisms, of which social support plays a key role.

Regarding work-associated environmental factors, exposure to violence was linked to increased burnout, this being supported by other literature data [86,87]. Adding violence prevention to larger organizational plans for employee well-being, like mental health assistance, systematic debriefing after events, and encouraging a culture of self-reporting, can make employees more resilient and less likely to burnout. Weng et al. [61] found that the EI development could be useful in preventing burnout, not only directly, but also mediated by less exposure to violence. Individual trainings in EI development, self-defense, and de-escalating techniques, alongside changes in logistics and workplace design (such as an increase in safety strategies and increased security), may be effectively employed to address or prevent burnout [88,89].

In terms of work stability, healthcare workers with temporary contracts are exposed to a higher risk of burnout because of higher perceived insecurity, but also as a result of often higher workload or fewer paid hours. Personal resources, such as resilience and EI, could act as compensating factors in such work conditions. Their positive role is also reported among employees who have a stable job but feel like caught in a trap, with more administrative tasks, less meaningful work time, and without any exit options [90].

Since both workload and dissatisfaction with career choices were found to increase burnout, while positive work experience might have a protective role, organizational interventions may prove to be of critical importance. Alongside the aforementioned mentorship programs for residents, an early target component might be vocational assistance, in order to help with early career dissatisfaction.

### 4.2. Protective Role of EI

Trait/self-report emotional intelligence (EI) tests assess individuals’ self-perceptions of their emotional capabilities and include affective dispositions such as self-control, adaptability, and emotion regulation, which are directly pertinent to managing stress and emotional demands. The same self-report methodology employed in numerous burnout assessments may also enhance the observed relationships. On the other hand, ability-based EI tests, like tests of how well someone can understand and manage emotions, are less affected by personality traits and self-perceptions. They may have a more indirect effect on burnout by improving problem-solving and interpersonal effectiveness. As a result, correlations may be more pronounced for trait/self-report emotional intelligence than for ability emotional intelligence.

In terms of the correlation between burnout and EI, our selected studies identified an inverse relationship, which was identified in the existing literature not only for EI as a whole, but also for its distinct subcomponents (well-being, self-control, emotionality, and sociability) [91]. Specifically, Mitra et al. [62] pointed out that burnout had a significant negative correlation with EI’s well-being component and a positive correlation with self-control and sociability. Among distinct EI components, self-control seems to have an important role, as it is connected to adaptability in changing environments, but also with personal values and personal accomplishments [92]. The study conducted by Jahanazeb et al. [63] claims that self-control has a direct inverse relationship to burnout, with higher self-control ensuring a better control of emotional reactions to stressors, which, in turn, lowers the risk of burnout. Encouraging self-control (via therapies like mindfulness training, cognitive–behavioral methods, and programs that boost emotional intelligence) may not only be a valuable strategy to prevent or delay burnout, but also to impede mental illness resulting from the failure of emotional self-regulation mechanisms. The inclusion of such programs seems to have substantial benefits among healthcare students and residents [40,43,93,94]. About one-third of the research took place during or after the COVID-19 pandemic, indicating that stresses associated with the pandemic may partially explain the diversity in burnout–emotional intelligence relationships. Longitudinal data, when available [40,50,58,66,68], indicate that higher baseline emotional intelligence (EI) correlates with reduced subsequent burnout after controlling for relevant factors. Conversely, it is also plausible that prolonged emotional exhaustion may diminish perceived EI or weaken emotional regulation capabilities over time. In addition, unquantified contextual variables—such as workload, staffing ratios, leadership methodologies, moral discomfort, and recurrent exposure to suffering—may obscure discerned correlations. These factors highlight the necessity for prospective, controlled research to elucidate causal pathways and guide focused interventions.

### 4.3. Factors Influencing EI

The gender-related influences regarding EI levels in the identified articles showed either higher EI in women or men, or did not find any correlation of EI with gender or age. This is explained, as in the case of the relationship gender–burnout, as being possibly influenced by cultural differences, particularly by feminine stereotypes, potentially obscuring the role of gender. In the current literature, many studies were inconclusive about which gender is more EI-privileged, although the association of gender with EI seemed significant. Some studies in the current literature [95,96] reported higher EI in women, while, on the other hand, Almeneessier et al. [44] found higher EI in men. The higher number of articles reporting EI as more prevalent in women may result from the dimensions of EI tightly related to “feminine” stereotypes (such as emotional attention, emotional regulation, and emotional clarity), therefore potentially creating a self-reporting bias [97]. In order to address this taxonomy-induced misinterpretation, future studies could use gender-neutral language in the self-reported questionnaires.

Regarding the connection between age and EI, the identified articles found inconsistent associations, with some of them [40,56] stating that either higher or lower age was related to higher EI, while others [41,42] claimed that age plays no role. The study conducted by Cabello et al. [95] found that EI levels varied with age according to a U-inverted curve, with younger and older adults scoring lower than middle-aged adults. Another study conducted by Fariselli et al. [98] concluded that several components of EI increase with age, whereas others do not, further raising the issue of the selection of the EI components potentially developing through training. These findings emphasize the importance of future research focused on specific EI domains and their development in relation to other variables.

The studies included in this review identified a positive correlation between high EI and higher income. This could be important at least from two perspectives: firstly, the choice of EI-developing strategies could be influenced by the existing outcome, thereby limiting the access of low-income individuals to all kinds of programs of EI development. Secondly, EI could predict income itself, making its development a path to better societal rewards. This hypothesis is backed up by the results of a recent meta-analysis by Sanchez-Gomez et al. [99], who found that, after taking all the other variables into consideration, participants who displayed higher levels of EI and emotional-repair capacities had higher salaries, making EI one of the relevant variables when talking about building professional success.

The included papers suggested a positive relationship between sleep, exercise, and EI. This is consistent with the results of Killgore et al. [100], who found that sleep duration and habitual sleep quality are both independently associated with self-perceived dispositional aspects of EI. These benefits are further supported by the study by Sepdanius et al. [101], who also found a positive correlation between sleep quality and EI. Acebes-Sánchez et al. [102] found a gender-specific positive effect of physical activity, associated with higher emotional clarity and emotional repair in men and higher emotional attention in women. These findings could be the subject of a series of chain-related individual interventions. Therefore, by tackling the development of healthy habits such as the ones described, not only could the individual improve EI levels, but ultimately, indirectly prevent burnout.

## 5. Limitations and Future Directions

This study has several limitations. It was run as a narrative review, so some relevant articles may have been excluded. This review was limited to peer-reviewed journal articles published in English, which may introduce language and publication bias; the grey literature was not included. Duplicate sample risk was minimized by screening author lists, affiliations, and study periods; where overlaps were suspected, the most complete dataset was retained. This review was not preregistered; however, eligibility criteria, search strategy, and data extraction procedures were defined a priori to minimize hindsight bias. Other limitations were derived from the heterogeneity of scales used for evaluating burnout and EI, the self-reporting method, and the heterogeneity of the cultural backgrounds of respondents and the variations in study quality. Most included studies relied on self-reported EI and burnout instruments, which may introduce common-method bias and inflate associations. Although the general focus was on health professionals, these studies included medical personnel from different categories and specialties as participants, each of them having particular burnout and EI characteristics. Future research should address these limitations through preregistered, longitudinal, and multicenter designs employing standardized or ability-based EI instruments. Comparative analyses across professional groups would clarify whether certain specialties benefit differently from EI-based interventions. Mechanistic studies are required to elucidate how specific the EI traits interact with distinct burnout dimensions. Furthermore, intervention studies in addressing burnout—such as mindfulness-based stress reduction [103] and EI-enhancing training—should evaluate not only clinician well-being but also organizational outcomes (e.g., absenteeism, turnover) and patient-related endpoints (e.g., satisfaction, quality of care).

## 6. Conclusions

The current paper had the main goal to analyze the connection between burnout and EI in healthcare professionals. During this research, it became increasingly obvious that burnout syndrome has evolved to become a significant concern for employees in the healthcare sector. In this context, the identified protective role of EI is important because it suggests EI as both a potential target and a promising instrument against professional exhaustion; therefore, employing EI-enhancing strategies could help address the root causes of burnout. Furthermore, expanding our understanding of this connection could offer a theoretical framework that might prove useful in redefining strategies in high-stress fields. Among the determinants of EI, many may be integrated into individual and organizational interventions. Although the current study was not specifically focused on evaluating the comparative efficacy of direct vs. indirect (e.g., EI-enhancing) burnout-reducing strategies, this topic is very attractive for future research, considering its potential for building cost-effective systems of psychological support for healthcare professionals, reducing turnover, and guiding organizational policies in healthcare.

## Data Availability

No new data were created or analyzed in this study.

## Supplementary Materials

The following supporting information can be downloaded at https://www.mdpi.com/article/10.3390/healthcare13172156/s1, Table S1: Full-text articles excluded with reasons (n = 7).

## Abbreviations

The following abbreviations are used in this manuscript:

EI	Emotional Intelligence
EE	Emotional Exhaustion
DP	Depersonalization
PA	Personal Accomplishment
PO	Psychological Ownership
