# Supplementary material for: Contributing Factors to Burnout in Healthcare Professionals—Does Emotional Intelligence Play a Protective Role? A Narrative Review
Source: Healthcare (Basel). 2025 Aug 29;13(17):2156. doi: 10.3390/healthcare13172156 (PMC12428771; doi:10.3390/healthcare13172156)
Supplement: Supplementary file 1 [file healthcare-13-02156-s001.zip › healthcare-3801182-supplementary.pdf]

Supplementary Materials

The 7 excluded articles not meeting inclusion criteria are listed in Table S1 along with reasons for exclusion.

Table S1. Full-text articles excluded with reasons (n = 7).

No.	Reference	Country / Setting	Reason for Exclusion
1	McKinless E. Impact of stress on nurses working in the district nursing service. Br J Community Nurs. 2020;25(11):555–561. doi:10.12968/bjcn.2020.25.11.555	UK	Focused only on stress, did not evaluate burnout explicitly
2	Sabo B. Reflecting on the concept of compassion fatigue. Online J Issues Nurs. 2011;16(1):1. doi:10.3912/OJIN.Vol16No01Man01	USA	Did not directly correlate with EI
3	Kurnianto DK, Ariqoh AS, Hasian FP, Nugroho II, Nisfullaili J, Damayanti RW. Factors that Affect the Job Performance of the Health Workers in Indonesia. Springer; 2022.	Indonesia	Studied both concepts but not their connection
4	Filipponi C, Pizzoli SF, Masiero M, Cutica I, Pravettoni G. Trait EI and compassion fatigue in healthcare professionals. Psychol Rep. 2024;127(2):868–886.	Italy	Studied compassion fatigue, not burnout
5	Maina RN. The Relationship Between Burnout and Self-Awareness Among Emergency Healthcare Workers.	Kenya	Studied only self-awareness, not full EI
6	Lai IJ, Liao LL, Lee CH, Li PS. Work stress, EI, and emotional exhaustion in dietitians. Taiwan Gong Gong Wei Sheng Za Zhi. 2018;37(4):464.	Taiwan	Correlated EI only with emotional exhaustion, not full burnout
7	Liu L, Xu P, Zhou K, Xue J, Wu H. Mediating role of emotional labor in EI–fatigue association among doctors. BMC Public Health. 2018;18(1):881.	China	Assessed fatigue, not full burnout
